# Comparative analysis of long DNA sequences by per element information content using different contexts

**DOI:** 10.1186/1471-2105-8-S2-S10

**Published:** 2007-05-03

**Authors:** Trevor I Dix, David R Powell, Lloyd Allison, Julie Bernal, Samira Jaeger, Linda Stern

**Affiliations:** 1 Faculty of Information Technology, Monash University, Clayton, 3800, Australia; 2 Victorian Bioinformatics Consortium, Monash University, Clayton, 3800, Australia; 3 Computer Science and Software Engineering, University of Melbourne, Melbourne, 3010, Australia

## Abstract

**Background:**

Features of a DNA sequence can be found by compressing the sequence under a suitable model; good compression implies low information content. Good DNA compression models consider repetition, differences between repeats, and base distributions. From a linear DNA sequence, a compression model can produce a linear information sequence. Linear space complexity is important when exploring long DNA sequences of the order of millions of bases. Compressing a sequence in isolation will include information on self-repetition. Whereas compressing a sequence Y in the context of another X can find what new information X gives about Y. This paper presents a methodology for performing comparative analysis to find features exposed by such models.

**Results:**

We apply such a model to find features across chromosomes of *Cyanidioschyzon merolae*. We present a tool that provides useful linear transformations to investigate and save new sequences. Various examples illustrate the methodology, finding features for sequences alone and in different contexts. We also show how to highlight all sets of self-repetition features, in this case within *Plasmodium falciparum *chromosome 2.

**Conclusion:**

The methodology finds features that are significant and that biologists confirm. The exploration of long information sequences in linear time and space is fast and the saved results are self documenting.

## Background

The paper presents a methodology for exploring long DNA sequences, of the order of millions of bases, by means of their information content. We bring together two of pieces of our work, a Bayesian compression model and a graphical exploration tool, and give examples illustrating the methodology.

Compression is used to find the features of a sequence and common features that relate one sequence to another. Linear information content sequences are then used to locate various kinds of common information. Genomic subsequences or regions identified through this process can then be further investigated.

The compression problem is to calculate the information content per base, producing an *information sequence*. Information is relative, i.e. it depends on the context. The context can include one or more other sequences and hence information content can *relate *two or more sequences.

Note that an information sequence is 1-dimensional; operations such as difference, zoom, smooth and threshold are efficient, taking linear time and space. This is in contrast to the traditional 2-dimensional plots of one sequence against another which must be stored at low resolution for long sequences.

Any per element compression model can be used to create an information sequence. Here we use our Approximate Repeats Model (ARM) [1-3], however, other statistical models that produce an information sequence could be used. We present the ARM, introduce our tool to manipulate information sequences, and explore its use for the red alga *Cyanidioschyzon merolae *[4] and the malaria strain *Plasmodium falciparum *[5].

## Methods

### DNA sequence compression

We wish to examine the information content of sequences. Information content and compressibility are inherently related: low information content implies high compressibility and high information content implies low compressibility. So, if one has an efficient encoding of a sequence, then it can be argued that one has a good model of that sequence. From Shannon [6] we know that an efficient encoding is related to its probability by the log likelihood. That is, information *I*(*m*) = -*logP*(*m*), where *P*(*m*) is the probability of *m *occurring.

When trying to make an inference from some data using a Bayesian technique, we attempt to maximize the posterior probability, *P*(*H*|*D*) = *P*(*D*|*H*) × *P*(*H*)/*P*(*D*) for hypothesis *H *and data *D*. If our model (hypothesis) has a nuisance parameter about which we do not care to make an inference, we should sum over all possible values for this parameter. This is necessary when using sequence alignment to infer how related two sequences are. If we are only interested in whether the sequences are related or not we should sum over all possible alignments [7].

The way that compression models for DNA handle repetition can be broadly classified as substitutional or statistical. A *substitutional *model uses some form of pointer back to an earlier instance of a repeated subsequence to encode a later instance. On the other hand, a *statistical *model encodes the sequence element by element using a probability distribution over the possible values of the next element in the sequence. The distribution can be formed as a blend of opinions derived from the base distribution and from the length and fidelity of matches between recent history and earlier parts of the sequence. A statistical method can directly yield a per element information sequence, in addition to deriving a compressed encoded sequence. However, there is no simple natural way to derive a per element information sequence for a substitutional model.

Significant advances in substitutional compression models for DNA include: BioCompress [8] and BioCompress-2 [9]; and the more recent DNACompress [10]. And for statistical models: Loewenstern and Yianilos [11]; Korodi and Tabus [12]; and Cao et al. [13] who also produce a per element information sequence. The Approximate Repeats Model (ARM) used here, and described in the next section, is at heart a substitutional model yet it behaves much like a statistical model.

It is worth noting at this point that not all applications of compression need the production of an information sequence. The encoded sequence may be sufficient. And sometimes just the length of the encoded sequence may be enough, for example when searching for the best in a class of models. However, our work here requires a per element information sequence.

### Approximate Repeats Model

Here we choose to use the Approximate Repeats Model (ARM) [1] to provide per element information sequences. Any good per element compression scheme could be used. The ARM is designed to compress DNA sequences well. Compression values given in [13] and [1] show that the ARM is rarely bettered on common data sets and then only marginally. It is a Bayesian model that applies minimum-message-length inference [14].

DNA sequences often have regions that are highly similar, with only a few differences. Given the double-stranded nature of DNA, it is also common for DNA to contain reverse-complementary repeats – sometimes called palindromes – due to complementary matching in the reverse direction of A to T, C to G and vice versa. The ARM compresses a sequence by finding each region that is similar to a previously encountered region and encoding it as "similar to this other region, but with these changes". It also looks at the reverse-complement of the sequence so far to find similarities. (An implementation of the model is available [15].)

The ARM considers a DNA sequence a base-pair (bp) at a time from left to right. Each bp may have originated in one of two ways:

1. It may have been generated from a *base model*. This base model can in principle be any sequence model. We have typically used a low-order Markov Model.

2. The bp may have been generated as part of a repeated region. A repeated region is specified by first giving the position in the sequence where this region is repeated from; a uniform distribution is used to encode this position.

The description to this point is quite similar to the Ziv-Lempel [16] algorithm. The difference is in how a repeated region is treated: Each bp from a repeated region may be copied, deleted or changed, or a bp may be inserted. The length of a repeat is encoded using a geometric distribution; while this may not be ideal, it allows for a more efficient algorithm.

Notice that this method of treating repeated regions is very similar to the way local-alignment algorithms [17] are used to model sequence variations. This is quite deliberate, the ARM is in effect aligning a sequence against the sequence already seen. It achieves good compression in regions that would have good alignment scores. The implementation of the ARM supports both simple gap costs and affine gap costs. It is possible to view a two-dimensional plot of the self-alignment used in the ARM but such an image is a very coarse way to look at the results. For example, for a sequence of a million elements, each pixel in the image would represent roughly one thousand bases. Thus it is necessary to find a better way to deal with the compression results, we suggest using a 1-dimensional plot of the information content.

The per element information content for a sequence under the ARM is formed by a Bayesian blend of all possible explanations for the current element. Outside of repeat regions, the base model provides the most probable explanation. As an approximately repeated region starts to be matched, the base model is still the most probable and the repeat carries little weight. As more of the repeat region is matched, its contribution increases providing a relatively smooth transition in the information sequence.

Often there are many competing sequence alignments that are almost equally good. This also happens within the ARM. A region may be quite similar to a number of earlier regions and we do not want to pick just one of them to copy from. These repeated regions may be treated as mutually-exclusive hypotheses, and since we do not care to make an inference about which one is the best, we sum over all of their probabilities, in effect removing a nuisance parameter. This also allows the ARM to trade-off the frequency and length of a repeat against its (in)fidelity.

The ARM has a small number of parameters – probabilities for the beginning of a repeat, for the possible mutations and for ending a repeat. An iterative EM algorithm is used to converge on the best set of parameter values: First, the ARM is used with some initial values for these parameters. Then the results from applying the model are used to estimate new values for the parameters. These new parameters replace the initial values and this two-step process is iterated until it converges.

### 1-D information content viewer

**Figure 1:**
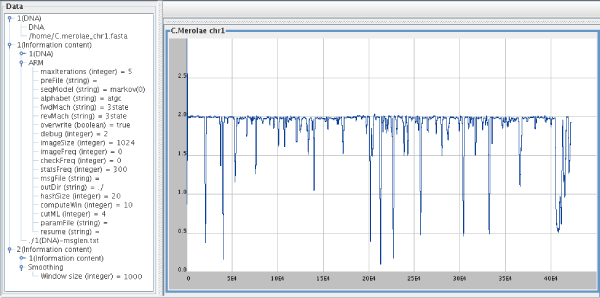
**Plot for C. merolae chromosome 1, smoothing window 1000**. Information sequence from ARM for *C. merolae *chromosome 1, with a smoothing window of 1000 bp.

**Figure 2:**
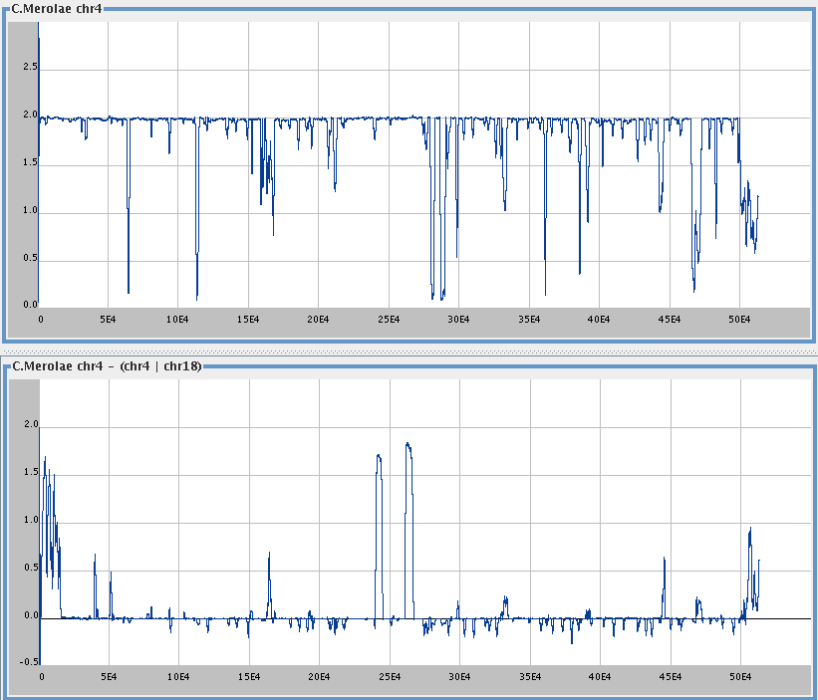
**Plot for C. merolae chromosome 4 and its difference from chromosome 4 given 18, smoothing window 1000**. Information sequence from ARM for *C. merolae *chromosome 4 at top; and the resulting information sequence after subtracting information sequence for chromosome 4 given 18. Smoothing windows of 1000 bp.

InfoV is a Java platform used to explore the structure of sequences using arbitrary compression models. It provides functionality to import biological sequences such as DNA, use compression models to generate information content sequences, and interactively display multiple plots for the analysis. This tool also provides various functions to manipulate sequences such as smooth, cut, append, calculate the difference between numeric sequences, and find the reverse complement of DNA sequences. Additionally, InfoV annotates how sequences are derived; this includes the storage of the model parameters and functions used to create sequences. Figure 1 illustrates the displays for the compression of chromosome 1 of *Cyanidioschyzon merolae *alone; the troughs showing self repetition. However, InfoV is particularly useful for performing comparisons in different contexts, such as in figure 2 where a difference plot is used to highlight information, at the peaks, contributed by the context. These figures are discussed in the next section.

The current implementation of InfoV is focused on DNA sequences and includes the ARM. However, it has a generic, extensible design, which enables the analysis of other type of sequences, such as character and numeric sequences, and the use of other compression models.

## Results and discussion

We applied the ARM to find approximate repeats within each of chromosomes 1, 2, 3, 4, 5, 6, 11, 12, 16 and 18 of *C. merolae *[4] and between pairs of chromosomes. The 1-d information content graph, *I*(*c*1), is given in figure 1 for chromosome 1. It has been smoothed, displaying the average of a 1000 wide sliding window. We can easily store the whole graph and dynamically explore the low information areas. The window size should be of the order of the feature being searched for. Typically, one looks for large features first. The viewer facilitates zooming-in and re-smoothing with smaller window size, to either further investigate regions or to find smaller features. Subsequences of interest can be saved to file for further investigation starting, say, with a Blast search. This figure also shows the history window for the plot.

Figure 2 shows *C. merolae *chromosome 4 compressed alone. The figure also contains a difference plot of the information content for chromosome 4 alone minus that for chromosome 4 given 18, i.e. *I*(*c*4) - *I*(*c*4|*c*18). To calculate the information sequence *I*(*c*4|*c*18), the ARM prepends chromosome 18 to 4, and thus compresses chromosome 4 in the presence of chromosome 18. This shows explicitly what new information content chromosome 18 brings concerning chromosome 4.

In this case, we find repeated regions from 239406 to 244000 corresponding to 974903 to 970308 in chromosome 18, and another from 260529 to 265988 corresponding to 961910 to 967371 in 18. The first region is a probable myo-inositol 2-dehydrogenase [18] (gene CMR475C) and the second contains a hypothetical protein.

Importantly, all of these plots are 1-dimensional. They can be computed at full resolution and stored, even on a small computer. We used the ARM but the same can be done for any (your favourite) statistical compression model. Common operations such as difference, smooth, zoom and threshold can be performed quickly in linear time. A difference plot shows what *new *information the addition of a context tells us about a sequence; features already revealed by the original context, here chromosome 4 alone, are discounted by the difference.

**Figure 3:**
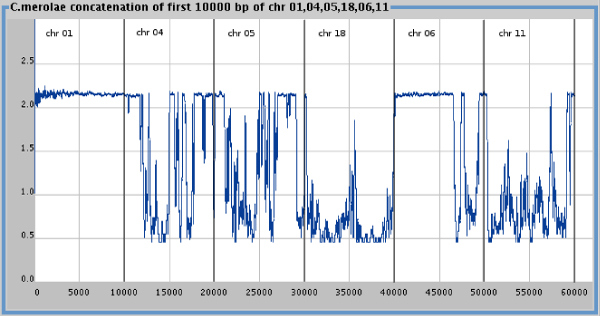
**Plot for C. merolae 10000 bp for chromosomes 1, 4, 5, 18, 6 and 11, smoothing window 100**. Information sequence from ARM of the (concatenated) initial subtelomeric regions (10000 bp) of *C. merolae *chromosomes 1, 4, 5, 18, 6 and 11. Very little self repetition for chromosome 1. Here chromosome 6 compresses due to previous contexts, but alone has very little self repetition. Smoothing window of 100 bp.

We also investigated the subtelomeric regions of *C. merolae*. Pairwise comparisons *I*(*c*_*i*_|*c*_*j*_) confirmed known results [4]. We summarize the results in figure 3 showing that the subtelomeric regions for chromosomes 1, 4, 5 and 18 belong to element P and those for chromosomes 6 and 11 belong to element PH. Notice that chromosomes 1 and 6 do not compress well in their contexts.

**Figure 4:**
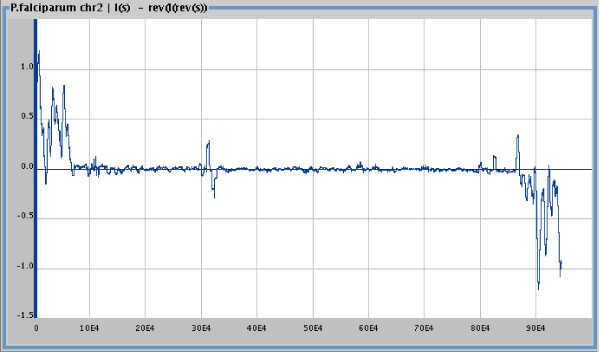
**Plot of *I*(*c*2) - *rev*(*I*(*revcomp*(*c*2))) for chromosome 2 of P. falciparum, smoothing window 5000**. Sequence highlighting the first and last reasonably long repeats within *P. falciparum *chromosome 2. The right to left information sequence is found for the reverse complement and is then reversed to be left to right. The resulting sequence is subtracted from left to right information sequence.

Our final example is for chromosome 2 of *P. falciparum *[19]. The *P. falciparum *genomic sequence is approximately 80% AT rich. It should be noted that the base Markov model and the repeat-region model within the ARM are not troubled by this bias which is shared by both the source and target of a repeat and hence cancels out without causing false positive signals. Information sequences derived by the ARM are directional. To this point, only left to right sequences have been derived. Figure 4 shows a difference plot of *I*(*c*2) - *rev*(*I*(*revcomp*(*c*2))) where *revcomp *gives the reverse complement of a DNA sequence and *rev *simply reverses the resulting information content sequence. The sequence from the first term is computed left to right; the second is computed right to left and then reversed. Such difference plots highlight the first and last instances of approximate repeated subsequences.

Most of this difference plot gives values close to zero. But at both ends there are large differences from the baseline reflecting the known repetitive structure of chromosome ends for *P. falciparum*. The differences in sign are just a result of reversal and subtraction. Telomere-associated repeat elements include Rep20, and the var, rif and stevor genes that are involved in its virulence [20].

The above examples illustrate how to use linear information sequences to highlight similarities within a genomic sequence, including the first and last occurrences, and to find similarities in the context of other sequences. This is the basis of our methodology for exploring long DNA sequences.

Smoothing derived information sequences is an integral part of the process. Information sequences can be quite busy without smoothing. Window sizes of roughly the size of what is sought are necessary. Typically, one starts with a large window size which is successively reduced as more detail is investigated.

The methodology for comparing long DNA sequences by information content is as follows:

1. Look for repeat regions from *I*(*c*). Find the first instances of repeats as well using *I*(*c*) - *rev*(*I*(*revcomp*(*c*))).

(a) Zoom in and capture interesting (compressible) regions for further investigation.

(b) Reduce the smoothing window size to find smaller repeat regions.

2. Repeat the above applying different contexts using *I*(*c*) - *I*(*c*|*ctx*).

## Conclusion

Information is relative to what is known. A sequence *Y *can be compressed firstly in a context *ctx*1 and then in a context *ctx*2 where *ctx*2 is *ctx*1 plus a sequence *X*. The difference between the information sequences for *Y*|*ctx*1 and for *Y*|*ctx*2, i.e. *I*(*Y*|*ctx*1) - (*Y*|*ctx*2), shows the *new *information that *X *gives us about *Y*. Mere background statistical properties of *Y *and *X*, that were already known from *ctx*1 and/or *Y *itself, are discounted.

We have shown how to use 1-dimensional information sequences derived from long DNA sequences for the comparison of a sequence with itself and with additional contexts. A methodology has been outlined to identify sequence similarities for subsequent investigation. Importantly, exploration of full-resolution information sequences is carried out in linear time and space. The information sequences can be computed from within our tool, or computed off-line and imported.

## Authors' contributions

LS ran the ARM over *P. falciparum *and investigated the resulting information sequences. SJ ran the ARM over the *C. merolae *data and investigated the resulting information sequences. JB developed the code to the information content viewer. LA developed the ARM and the use of associated contexts, and the methods to investigate information sequences. DP redeveloped the code for the ARM and contexts, and developed the viewer. TD developed the ARM, methods to investigate information sequences, and the information content viewer. All authors read and approved the final manuscript.
